# Precise Species Identification and Taxonomy Update for the Genus *Kluyvera* With Reporting *Kluyvera sichuanensis* sp. nov.

**DOI:** 10.3389/fmicb.2020.579306

**Published:** 2020-09-16

**Authors:** Lina Liu, Yu Feng, Li Wei, Fu Qiao, Zhiyong Zong

**Affiliations:** ^1^Center of Infectious Diseases, West China Hospital, Sichuan University, Chengdu, China; ^2^Center for Pathogen Research, West China Hospital, Sichuan University, Chengdu, China; ^3^Division of Infectious Diseases, State Key Laboratory of Biotherapy, Chengdu, China; ^4^Department of Infection Control, West China Hospital, Sichuan University, Chengdu, China

**Keywords:** *Kluyvera sichuanensis*, *Kluyvera*, genome sequences, sinks, taxonomy

## Abstract

*Kluyvera* is a genus within the family *Enterobacteriaceae* and can cause various human infections but remains poorly studied. A carbapenem-resistant *bla*_NDM–__1_-carrying *Kluyvera* strain 090646^T^ was isolated from a hospital sink in Chengdu, Sichuan province, China. Whole genome sequencing of the strain revealed that it had 28.2 to 42.3% *in silico* DNA-DNA hybridization (*is*DDH) scores and 84.15 to 90.10% average nucleotide identity (ANI) values with other *Kluyvera* species. Both values are well below the ≥ 70.0% *is*DDH and ≥ 95–96% ANI cutoffs to define bacterial species, suggesting that the strain represents a novel species of the genus *Kluyvera*, for which the name *Kluyvera sichuanensis*. nov. is proposed. Type strain of *K. sichuanensis* is 090646^T^ (=GDMCC 1.1872^T^ =KCTC 82166^T^). Strain 090646^T^ can be differentiated from other *Kluyvera* species by its ability to utilize D-sorbitol but not β-galactosidase (ONPG), D-mannose, glycerin, raffinose, nor sucrose. There were 47 genome sequences labeled as *Kluyvera* in GenBank, which were curated for precise species identification. Only 33 of the 47 genomes were indeed of *Kluyvera* and four novel *Kluyvera* genomospecies were identified, highlighting that the species assignation of bacterial genomes in GenBank need to be curated. Genome sequencing for more strains is required to understand the genus *Kluyvera*.

## Introduction

*Kluyvera* is a genus of the family *Enterobacteriaceae* (Farmer et al., 1981) and can be found in soil, water, sewage, and healthcare environment (Farmer et al., 1981; Li et al., 2019; Mutoh et al., 2019). In human, *Kluyvera* strains appear to be largely colonized in the gastrointestinal tract but also cause a wide range of infections such as bacteremia, cholangitis, diarrhea, neonatal meningitis, peritonitis, and pneumonia with severity from mild to fatal diseases (Sarria et al., 2001; Lee et al., 2019). Although *Kluyvera* is of clinical significance, it remains poorly studied. At the time of writing, the *Kluyvera* genus consists of only four species: *Kluyvera ascorbata*, *Kluyvera cryocrescens*, *Kluyvera georgiana*, and *Kluyvera intermedia* (Farmer et al., 1981; Muller et al., 1996; Pavan et al., 2005). Of note, the previously known *Kluyvera intestini* (Tetz et al., 2017) has been moved to the genus *Metakosakonia* in 2017 (Alnajar and Gupta, 2017). In this study, we first reported a novel *Kluyvera* species by examining the taxonomy and characterizing phenotypes of a strain from a handwashing sink. Then, we curated the species assignation of all *Kluyvera* genomes (*n* = 47) in GenBank and found 4 novel *Kluyvera* genomospecies based on genome analysis. Last, we provided an updated taxonomy for the genus *Kluyvera* as a reference for future studies.

## Materials and Methods

### Strain Isolation and Identification

Strain 090646^T^ (also called SCKS090646) was recovered from the residual water of a handwashing sink as part of an infection control surveillance program on sinks. The residual water was sampled using a sterile rayon swab (Copan; Brescia, Italy) moistened with tryptic soy broth (TSB) (Hopebio, Qingdao, China). The swab was placed into a 15 ml sterile tube containing 6 ml TSB and was incubated at 37°C overnight. Then the tube was centrifuged, and supernatant was discarded, and precipitants were resuspended with 1 ml TSB. A 50 μl suspension was streaked onto a CHROMagar Orientation colorimetric plate (Chromagar; Paris, France). Isolates from sinks were subjected to preliminary species identification using matrix-assisted laser desorption ionization time of flight mass spectrometry (MALDI-TOF; Bruker, Billerica, MA, United States) with a database version DB5989 according to the manufacturer’s instructions.

### Analysis of the 16S rRNA Gene Sequence

**FIGURE 1:**
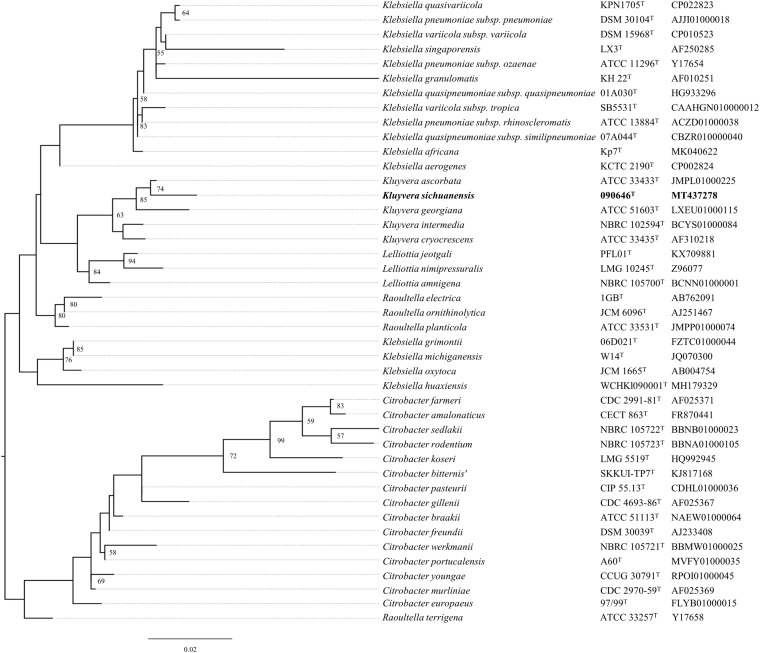
Phylogenetic tree of *Kluyvera sichuanensis* 090646^T^, other *Kluyvera* species and other closely related genera based on 16S rRNA gene sequences. The tree was inferred using the maximum-likelihood method. Bootstrap values >50% (based on 1,000 resamplings) are shown. Bar, 0.02 substitutions per nucleotide position.

A nearly complete sequence (1,411 bp) of the 16S rRNA gene was obtained from strain 090646^T^ by PCR as described previously (Moreno et al., 2002). The corresponding 16S rRNA gene sequence of type strains of other *Kluyvera* species and other closely related genera sharing >97% nucleotide identity with that of strain 090646^T^ were retrieved from GenBank. The 16S rRNA gene sequences were aligned using Clustal Omega (Madeira et al., 2019) and a maximum-likelihood phylogenetic tree (Figure 1) was inferred by RAxML v8.2.12 (Stamatakis, 2014) under GTRGAMMA model with a 1,000-boostrap test.

### Phenotypic Characterization

Gram-staining reaction was performed as described previously (Smibert and Krieg, 1994). Cell motility was examined by observing the bacterial growth and diffusion on the deep semi-solid nutrient agar medium of 0.3% (w/v) agar (Hopebio, Qingdao, China). Anaerobic growth was examined by streaking the bacterial cultures on Brain Heart Infusion agar plates and placed in the GasPak^TM^ EZ Anaerobic Bag (BD; Franklin Lakes, NJ, United States) at 35°C for 3 days. After incubation in nutrient broth at 37°C for 3 days, flagella of strain 090646^T^ was observed with a H-7650 transmission electron microscope (Hitachi; Tokyo, Japan) as described previously (Hanada et al., 2002). Briefly, the centrifuged bacterial precipitation was fixed with 5% (v/v) glutaraldehyde and 1% (v/v) osmium tetroxide. Ultrathin sections of the sample were embedded in epoxy resin using a Reichert ultrathin microtome (AMETEK; Berwyn, PA, United States). The samples were stained with uranyl acetate and lead citrate and examined using the microscope.

The growth of strain 090646^T^ was examined in 5 ml aliquots of nutrient broth dispensed into tubes (16 mm, inner diameter) at temperatures of 4, 8, 18, 28, 32, 37, 42, 45, 48, and 50°C. Salt and pH tolerances were measured using nutrient broth at 37°C for 2 days at different NaCl concentrations (0.5, 1, 2, 3, 4, 5, 7.5, 10, and 15%, w/v) and at various pH values (with a pH unit of 4.0–12.0, in increments of 1.0 unit), respectively as described previously (Hu et al., 2017). Catalase activity test was conducted by examining the production of bubbles after addition of 3% (v/v) hydrogen peroxide solution, while oxidase activity was tested by using 1% tetramethyl-p-phenylenediamine dihydrochloride solution. DNase activity was detected with 1M HCl using DNase agar (Solarbio; Beijing, China) after 3 days of incubation at 30°C. Malonate, phenylalanine deaminase and potassium cyanide (KCN) experiments were performed using biochemical identification tubes (Huankai; Guangzhou, Guangdong, China). Commercially available API 20E, API 50CH, and API ZYM kits (bioMérieux; Marcy l’Etoile, France) were used for testing biochemical features and enzyme activities according to the manufacturer’s instructions with *Escherichia coli* strain ATCC 25922 and *Pseudomonas aeruginosa* strain ATCC 27853 as controls. All of the above experiments were performed in triplicate.

### Fatty Acid Analysis

The analysis of cellular fatty acids was performed by the Guangdong Institute of Microbiology (Guangzhou, Guangdong, China). Briefly, fatty acid methyl esters were extracted and analyzed by gas chromatography according to the instructions of the Sherlock Microbial Identification System (MIDI Inc.; Newark, DE, United States) as described previously (Sasser, 1990; Pandey et al., 2002). Peaks were automatically integrated, and fatty acid proportions were calculated using the MIDI identification database RTSBA6 (version 6.00; MIDI Inc.).

### Antimicrobial Susceptibility and Antimicrobial Resistance Genes of Strain 090646^T^

Minimum inhibitory concentrations (MICs) of amikacin, ampicillin, ampicillin-sulbactam, aztreonam, ceftriaxone, ceftazidime, cefepime, cefotaxime, cefuroxime, chloramphenicol, ciprofloxacin, colistin, imipenem, meropenem, piperacillin-tazobactam, sulfamethoxazole-trimethoprim, and tigecycline were determined using the microdilution broth method of the Clinical and Laboratory Standards Institute (CLSI) (CLSI, 2020). Breakpoints defined by CLSI (2020) for the *Enterobacteriaceae* were applied except for tigecycline, for which breakpoints defined by the European Committee on Antimicrobial Susceptibility Testing (EUCAST)^1^ were used. Antimicrobial resistance genes of clinical strain 090646^T^ were identified from genome sequences using the ABRicate program^2^ to query the ResFinder database^3^.

### Whole Genome Sequencing and Species Identification Based on Genome Sequences

Genomic DNA of strain 090646^T^ was prepared using the QIAamp DNA mini kit (Qiagen; Hilden, Germany) and DNA sequencing libraries were prepared using the NEBNext Ultra II DNA Library Prep Kit for Illumina (NEB; Ipswich, MA, United States). Whole genome sequencing was performed using the HiSeq X10 Sequencer (Illumina; San Diego, CA, United States) with a layout in 150 bp paired-end and aiming for at least 200× depth of coverage. Adaptor sequences and bases of low quality at the end were trimmed from raw reads using Trimmomatic v0.39 (Bolger et al., 2014) invoked in the pipeline of Shovill v1.1.0^4^ with default settings. Reads that passed the quality control were downsampled to approximately 150× in depth before being assembled into a draft genome of 090646^T^ using SPAdes v3.14.0 (Bankevich et al., 2012) under “–isolate” model. Contigs shorter than 200 bp or with coverage lower than 2× were discarded in the final assembly prior to being assessed in QUAST v5.0.2 (Gurevich et al., 2013). Genome completeness and contamination were examined using CheckM (Parks et al., 2015) with the marker set of *Enterobacteriaceae*. The genome sequences were reported following recommendations of standards for describing a new taxonomy (Chun et al., 2018). Plasmid replicon type was determined using the PlasmidFinder tool available from the Center for Genomic Epidemiology^5^.

**FIGURE 2:**
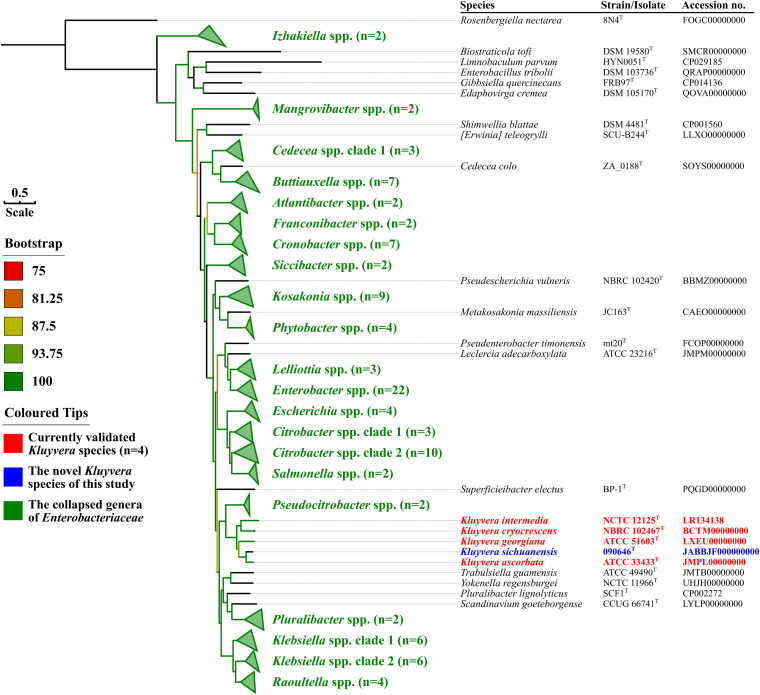
Maximum-likelihood phylogenomic tree of *Kluyvera sichuanensis* 090646^T^ and other species in the family *Enterobacteriaceae* based on the core genome. The four *Kluyvera* species known before this study are highlighted in red, while *K. sichuanensis* is shown in blue. Genera comprising multiple species are depicted in green with the number of species shown in parentheses. There are two clades for the genera *Cedecea*, *Citrobacter*, and *Klebsiella*. *Cedecea colo* belongs to a separate clade, while the remaining three *Cedecea* species (Supplementary Table S1) form *Cedecea* spp. clade 1. *Citrobacter* spp. clade 1 comprises *Citrobacter amalonaticus*, *Citrobacter rodentium*, and *Citrobacter sedlakii*, while the remaining 10 *Citrobacter* species (Supplementary Table S1) belong to *Citrobacter* spp. clade 2. *Klebsiella* spp. clade 1 comprises *Klebsiella aerogenes*, *Klebsiella africana*, *Klebsiella pneumoniae*, *Klebsiella quasipneumoniae*, *Klebsiella quasivariicola*, and *Klebsiella variicola*, while the remaining six *Klebsiella* species belongs to *Klebsiella* spp. clade 2. For genera or clade comprising only one species and *Kluyvera* species, the type strains and their nucleotide accession numbers are listed alongside the names of species. The tree was inferred using the maximum likelihood method under GTRGAMMA model with a 1,000-bootstrap test and branches with support over 75% are indicated by different colors. Bar, value indicates the nucleotide substitution per site.

Genome sequences of type strains of all *Enterobacteriaceae* species (Supplementary Table S1) were retrieved from GenBank and were annotated using Prokka v1.14.6 (Seemann, 2014). Single-copy genes shared by all genomes were identified using PIRATE v1.0.4 (Bayliss et al., 2019) with default settings and were defined as core genes (*n* = 684). Nucleotide sequences of core genes were aligned and concatenated using MAFFT v7.313 (Katoh et al., 2002) and AMAS v0.98 (Borowiec, 2016) prior to being fed into RAxML v8.2.12 (Stamatakis, 2014) for inferring a maximum-likelihood phylogenomic tree (Figure 2) under GTRGAMMA model with a 1,000-bootstrap test.

The average nucleotide identity (ANI), *in silico* DNA-DNA hybridization (*is*DDH), and the percentages of conserved proteins (POCP) values between strain 090646^T^ and type strains of all *Kluyvera* species were determined. ANI was determined using the JSpecies with a ≥95–96% cutoff for defining species (Richter and Rossello-Mora, 2009), while *is*DDH was performed using Genome-to-Genome Distance Calculator (formula 2) with a the ≥70.0% cutoff for defining species (Meier-Kolthoff et al., 2013). POCP was calculated as described previously (Qin et al., 2014).

### Curation of Species Identification for *Kluyvera* Genome Species in GenBank

**FIGURE 3:**
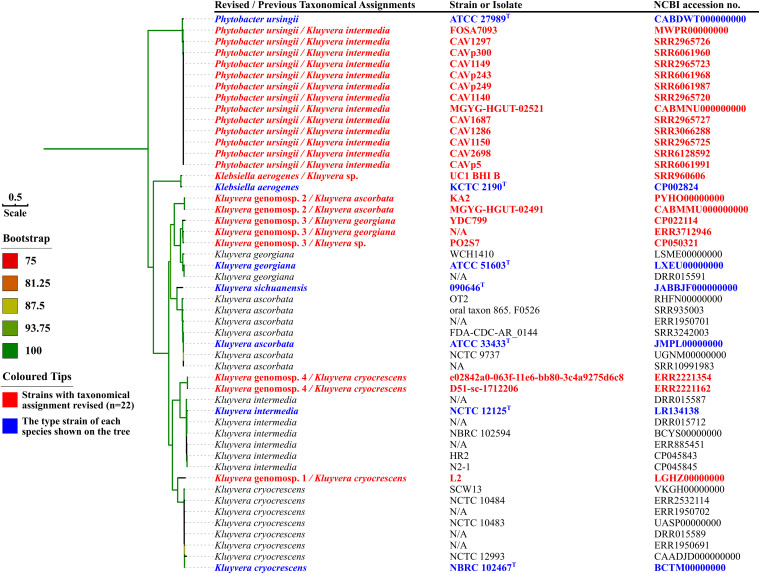
Maximum-likelihood phylogenomic tree of *Kluyvera sichuanensis* 090646^T^ and the 47 genomes labeled as *Kluyvera* based on the core genome. Strains and their nucleotide accession numbers are listed alongside the names of species. Type strains are depicted in blue. For genomes that are labeled as *Kluyvera* in GenBank but need to be revised as suggested in this study (depicted in red), the revised species names are shown first, while the current labels are shown after the slash. Genomosp. 1–4 are tentative *Kluyvera* species (Supplementary Table 3). The tree was inferred using the maximum likelihood method under GTRGAMMA model with a 1,000-bootstrap test and branches with support over 75% are indicated by different colors. Bar, value indicates the nucleotide substitution per site.

We searched the NCBI GenBank database and found 47 genome sequences labeled as *Kluyvera* including 20 assemblies and another 27 short-read data (Dataset S1 in the Supplementary, accessed by 10-06-2020). All of the 47 genome sequences were retrieved. Strains from NCBI collection with short-read data available were fed into the same pipeline as assembling strain 090646^T^ as described above. A maximum-likelihood phylogenomic tree based on core genes (Figure 3) was inferred for all *Kluyvera* genomes using RAxML v8.2.12 (Stamatakis, 2014) under GTRGAMMA model with a 1,000-bootstrap test as described above. All *Kluyvera* genomes were also subjected to precise species identification using ANI and *is*DDH as described above. Strains that have a <70% *is*DDH value and a <96% ANI value with any known *Kluyvera* species belong to a novel species, which is temporarily assigned genomospecies (genomosp.) here as the establishment of a novel species also requires phenotypic characterizations in addition to genome analysis.

### Comparative Genomic Analysis

Genomes labeled as *Kluyvera* and those of type strains of other species in *Enterobacteriaceae* (Supplementary Table S1) were annotated using Prokka v1.14.6 (Seemann, 2014) prior to being fed into PIRATE v1.0.4 (Bayliss et al., 2019) with 85% amino acid identity as the cutoff for comparative genomic analysis. Universal primers to amplify the complete sequence of the ferric reductase-encoding gene *fes* from all *Kluyvera* genomes were designed manually by examining the upstream and downstream sequences. Primers were checked for melting temperature (Tm), secondary structure, and primer dimer using oligoevaluator^6^. Virulence factors present in all *Kluyvera* genomes were determined using genome sequences to query the virulence determinants of the *Enterobacteriaceae* including *E. coli*, *Klebsiella pneumoniae*, *Salmonella enterica*, and *Shigella flexneri* in the VFDB database (Liu et al., 2019).

## Results

### Strain Identification

Strain 090646^T^ was recovered from the residual water of a handwashing sink at an ICU in Chengdu, China, on April 2019 as part of an infection control surveillance program on sinks. The strain was preliminarily identified as *Kluyvera ascorbata* by MALDI-TOF. A nearly complete sequence (1,411 bp) of the 16S rRNA gene of strain 090646^T^ has the highest identity with that of *Kluyvera ascorbata* ATCC 33433^T^ (99.22%) and is also highly similar with those of *K. intermedia* NCTC 12125^T^ (98.88%), *K. cryocrescens* NBRC 102467^T^ (98.58%), *K. georgiana* ATCC 51603^T^ (98.30%), *Klebsiella aerogenes* KCTC 2190^T^ (98.23%), *Raoultella terrigena* ATCC 33257^T^ (98.09%), *Citrobacter braakii* ATCC 51113^T^ (97.95%), and *Lelliottia amnigena* NBRC 105700^T^ (97.94%). In a maximum-likelihood phylogenetic tree (Figure 1) based on the 16S rRNA gene sequence alignment, strain 090646^T^ was allocated within the *Kluyvera* clade. However, strain 090646^T^ forms a relatively long branch separating from other *Kluyvera* species (Figure 1) and it is well known that analysis on 16S rRNA gene sequence is insufficient for accurate bacterial species assignation (Mulet et al., 2020). We therefore performed whole genome sequencing by HiSeq X 10 (Illumina; San Diego, CA, United States) for strain 090646^T^. A total of 1.42 gigabyte bases were generated, which were then assembled into 117 contigs (N50, 144,979 bp). The draft genome of strain 090646^T^ is 5,476,810 bp with a 54.51 mol% G + C content, which has been deposited at DDBJ/EMBL/GenBank under the accession JABBJF000000000.

A total of 684 core genes (Supplementary Dataset S2 in the Supplementary file) were identified in genome sequences of type strains of all *Enterobacteriaceae* species (Supplementary Table S1). In the maximum-likelihood phylogenomic tree based on core genes (Figure 2), strain 090646^T^ was also allocated within the *Kluyvera* clade. The POCP values between strain 090646^T^ and type strains of all *Kluyvera* species ranged from 90.81 to 93.18% (Table 1), suggesting that strain 090646^T^ indeed belonged to the genus *Kluyvera*. The ANI values between strain 090646^T^ and type strains of all *Kluyvera* species ranged from 84.15 to 90.10% (Table 1), lower than the ≥95–95% cutoff for defining species (Richter and Rossello-Mora, 2009). Consistently, *is*DDH values between strain 090646^T^ and type strains of all *Kluyvera* species ranged from 28.2 to 42.3% (Table 1), which are well below the ≥70.0% cutoff to define species (Richter and Rossello-Mora, 2009; Meier-Kolthoff et al., 2013). Both ANI and *is*DDH analyses clearly suggest that strain 090646^T^ represents a novel species of the genus *Kluyvera*. We proposed the species name as *Kluyvera sichuanensis* (si.chuan.en’sis. N.L. adj. *sichuanensis*, referring to Sichuan Province, China, where the type strain was recovered) after phenotypic characterization (see below). The type strain 090646^T^ has been deposited into Guangdong Microbial Culture Collection Center as GDMCC 1.1872^T^ and into the Korean Collection for Type Cultures as KCTC82166^T^.

**TABLE 1:** ANI, *is*DDH and POCP values between 090646^T^ and the type strains of other *Kluyvera* species.

Species	Strain	Accession number	ANI (%)	*is*DDH (%)	POCP (%)
*Kluyvera ascorbata*	ATCC 33433^T^	JMPL00000000	90.10	42.3	93.18
*K. cryocrescens*	NBRC 102467^T^	BCTM00000000	84.15	28.6	90.81
*K. georgiana*	ATCC 51603^T^	LXEU00000000	86.11	32.2	91.51
*K. intermedia*	NCTC 12125^T^	LR134138	84.26	28.2	91.18

### Phenotypic Characterization

We performed physiological and biochemical characterization for strain 090646^T^ (Table 2). The utilization of D-sorbitol combined with negative reactions for β-galactosidase (ONPG), D-mannose, glycerin, raffinose, nor sucrose is able to differentiate strain 090646^T^ from other *Kluyvera* species. Detailed results of the above tests are given in the species descriptions. The major cellular fatty acids of strain 090646^T^ are C_16__:__0_ (31.28%), C_17__:__0_ cyclo (17.56%), and summed in feature 8 (C_18__:__1_ω7c) (15.74%). By contrast, the composition of fatty acids in other *Kluyvera* species has not been reported.

**TABLE 2:** Biochemical characteristics of strain 090646^T^ and other *Kluyvera* species.

Characteristic	090646^T^	*Kluyvera ascorbata*	*K. cryocrescens*	*K. intermedia*	*K. georgiana*
Motility	+	+	+	+	+
Indole production	−	+	+	−	+
Voges–Proskauer reaction	−	−	−	+	−
Citrate utilization	+	+	+	+	+
H_2_S production	−	−	−	−	−
KCN growth in	+	+	+	+	+
Malonate utilization	+	+	+	+	−
NO_3_?NO_2_	+	+	+	+	ND
ONPG test	−	+	+	+	+
Oxidase	−	−	−	−	ND
Catalase	−	+	+	+	ND
Lipase	−	−	−	−	−
Deoxyribonuclease	−	−	−	−	−
Urease	−	−	−	−	−
Phenylalanine deaminase	−	−	−	−	−
Lysine decarboxylase	+	+	−	−	+
Arginine dihydrolase	−	−	−	−	−
Ornithine decarboxylase	+	+	+	+	+
Gelatinase	−	−	−	−	−
**Acid production from:**					
D-glucose	+	+	+	+	+
Sucrose	−	+	+	+	+
Dulcitol	−	−	−	+	+
D-sorbitol	+	−	−	+	−
Raffinose	−	+	+	+	+
Amygdalin	+	ND	ND	+	+
Glycerol	−	+	+	+	+
D-mannose	−	+	+	ND	ND
D-galactose	+	+	+	ND	ND
Esculin	+	+	+	+	+
methyl α-D-glucopyranoside	+	+	+	+	ND

*Data for other Kluyvera species are from references (Farmer et al., 1981; Pavan et al., 2005). Only key results of biochemical reactions are shown in the table. The following tests gave identical results for the five species: acid production from L-rhamnose (+), D-maltose (+), D-xylose (+), D-trehalose (+), D-cellobiose (+), D-melibiose (+), D-lactose (+), mannitol (+), salicin (+), L-arabinose (+), erythritol (−), D-arabitol (−), adonitol (−), inositol (−). ND, not done; +, positive; −, negative.*

### Antimicrobial Susceptibility and Antimicrobial Resistance Genes

Strain 090646^T^ was resistant to ampicillin, ampicillin-sulbactam, aztreonam, ceftriaxone, ceftazidime, cefepime, cefotaxime, cefuroxime, ciprofloxacin, imipenem, meropenem, piperacillin-tazobactam, but was susceptible to amikacin, chloramphenicol, colistin, sulfamethoxazole-trimethoprim, and tigecycline (Supplementary Table S2). Strain 090646^T^ had *bla*_NDM–__1_, encoding a metallo-β -lactamase to confer resistance to most β -lactams including carbapenems. Like strains of all other *Kluyvera* species, strain 090646^T^ harbors an intrinsic chromosomal *bla*_CTX–M_ gene, which encodes a CTX-M enzyme sharing the highest amino acid identity (90.72%, 264/291) with CTX-M-95 from *Kluyvera ascorbata*. Strain 090646^T^ also had *bla*_CTX–M–__14_, *bla*_OXA–__1_, *bla*_TEM–__1_, and *bla*_SFO–__1_ to mediate resistance to aztreonam, cephalosporins and penicillins, aminoglycoside-resistance genes *aac(6′)-Ib*-cr, *aac(3)-IId*, *aph(6)-Id*, and *aph(3″)-Ib*, fosfomycin-resistance gene *fosA3*, and quinolone-resistance gene *qnrS1*. Strain 090646^T^ contains seven plasmid replicons including IncFIB(pHCM2), RepA_pKPC-CAV1321, ColRNAI, Col(MGD2), ColpVC, Col440I, and Col440II. The association between antimicrobial resistance genes and plasmids warrants further investigations, which is, however, out of the scope of this study focusing on taxonomy.

### Species Curation for *Kluyvera* Genomes in GenBank

Except strain 090464^T^, there are 47 strains labeled as *Kluyvera* with genomes available in GenBank including 32 SRA and 20 assemblies (5 genomes with both SRA and assemblies) (Dataset S1, accessed by 10-06-2020). Among the 47 strains labeled as *Kluyvera*, 33 (70.2%, 33/47) were indeed of *Kluyvera*, while 13 strains actually belonged to *Phytobacter ursingii*, a non-*Kluyvera* species of the family *Enterobacteriaceae* (Pillonetto et al., 2018), and one strain was in fact of *Klebsiella aerogenes*. Twenty-five of the 33 *Kluyvera* strains could be correctly assigned to known *Kluyvera* species including *Kluyvera ascorbata* (*n* = 7), *K. cryocrescens* (*n* = 8), *K. georgiana* (*n* = 3), and *K. intermedia* (*n* = 7) including one strain labeled as *Kluyvera ascorbata* belonging to *K. georgiana* instead. No strain belongs to *K. sichuanensis* is found. The remaining eight strains were labeled as one of the four known *Kluyvera* species but actually do not belong to any known *Kluyvera* species. Instead, the 8 strains can be assigned to four novel *Kluyvera* genomospecies, genomosp. 1, 2, 3, and 4, based on ANI and *is*DDH values (Table 3) as well as illustrated in the phylogenomic tree (Figure 3). Species names are not assigned to the novel genomospecies here as they need to be characterized by phenotype methods. Currently, the *Kluyvera* genus therefore comprises nine species (Table 3). However, as there were only 34 *Kluyvera* genomes available, genome sequencing for more *Kluyvera* strains is required to further untangle the taxonomic composition of the genus. Collectively, nearly a half (*n* = 23) of the 47 *Kluyvera* genomes need to be curated for species assignations including species misidentification of 14 non-*Kluyvera* genomes and one *Kluyvera* genomes and update of 8 genomes to novel genomospecies.

**TABLE 3:** Updated Classification and nomenclature of the genus *Kluyvera*.

Species (*n* = 9)	Type strain	Genome accession no.		
*Kluyvera ascorbata*	ATCC 33433^T^	JMPL00000000		
*K. cryocrescens*	NBRC 102467^T^	BCTM00000000		
*K. georgiana*	ATCC 51603^T^	LXEU00000000		
*K. intermedia*	NCTC 12125^T^	LR134138		
*K. sichuanensis*	090646^T^	JABBJF000000		

**Genomospecies^a^**	**Reference strain**		**Closest species**	**ANI/*is*DDH,%**

Genomosp. 1	L2	LGHZ00000000	*K. cryocrescens*	89.93/38.4
Genomosp. 2	KA2	PYHO00000000	*K. georgiana*	87.87/33.3
Genomosp. 3	PO2S7	CP050321	*K. georgiana*	94.66/58.2
Genomosp. 4	D51-sc-1712206	ERR2221162	*K. intermedia*	90.15/39.1

*^a^Genomospecies refer to novel species for which species names have not been assigned as they need to be characterized by phenotype methods.*

### Virulence Factors

Strain 090646^T^ had multiple known virulence genes, i.e., the *chuS-U-W-X-Y*, *entA-B-C-E-F-S*, and *fepA-B-C-D-G* clusters, *fes*, *iutA*, and *shuV* for iron acquisition, *csgG* (encoding curli production assembly/transport protein), the *fimC-D-F-G-H* cluster (encoding type 1 fimbriae), the *kpsD-M-T* cluster (encoding K1 capsule), and *ompA* (encoding outer membrane protein A) (Supplementary Dataset S3 in the Supplementary). All of the virulence factors in strain 090646^T^ were also present in other *Kluyvera* genomes (Supplemenatry Dataset S3).

### Designing a Universal Pair of Primers for Differentiating *Kluyvera* Species by Comparative Genomics

As there are only few genomes for each *Kluyvera* species, we did not design a panel of primers targeting individual species but instead used a universal PCR approach. By comparative genomics, we identified that a ferric reductase-encoding gene *fes* was present in all *Kluyvera* genomes and had significant nucleotide variations for each *Kluyvera* species. We therefore designed a pair of primers (fes-up, 5′-TACGCTATTGCAAATGCAAA; fes-dw, 5′-TTGCATACCAAACTCCTGTC) able to *in silico* amplify the complete sequence (1,206 to 1,257 bp, Supplementary Table S3) of *fes* in all available *Kluyvera* genomes with <94% inter-species and >97% intra-species identities (Supplementary Dataset S4 in the Supplementary). Tm of the primers is 61.7 and 60.2°C, respectively, and therefore PCR with this pair of primers could be performed with a 55°C annealing temperature. Subsequent Sanger sequencing of the amplicons has the ability to differentiate all *Kluyvera* species with a >97% nucleotide identity cutoff. This approach warrants verification by experiments in the future.

## Discussion

In this study, we firstly described a novel *Kluyvera* species, *K. sichuanensis*, from a sink, using both genome and phenotypic analyses. The type strain 090646^T^ was recovered from a handwashing sink in an ICU, exhibited multi-drug resistance including resistance to carbapenems, and carried the *bla*_NDM–__1_ carbapenemase gene and therefore was of clinical significance.

We then curated all other *Kluyvera* genomes (*n* = 47) in GenBank and found the misidentification of 13 *P. ursingii* and one *Klebsiella aerogenes* of other genera as *Kluyvera*. This highlights that species identification for bacterial genomes in GenBank need to be carefully curated. Researchers who use bacterial genomes should be fully aware of the fact that the species labeled may be incorrect and therefore are recommended to check the precise species assignation by using genome-based analysis methods such as ANI and *is*DDH. In this study, we identified four novel genomospecies from the genome sequences in GenBank and therefore at present the *Kluyvera* genus comprises nine species (Table 3). However, as there were only 34 *Kluyvera* genomes available, genome sequencing for more *Kluyvera* strains is required to further untangle the taxonomic composition of the genus.

## Conclusion

In conclusion, a novel *Kluyvera* species, *K. sichuanensis* sp. nov., is identified and is characterized by both genome analysis and phenotypic methods. Four novel genomospecies are also identified, which warrant further phenotypic characterization. Genome sequencing for more *Kluyvera* strains is required to understand the genus of clinical relevance.

## Descriptions

### Description of *Kluyvera sichuanensis* sp. nov.

*Kluyvera sichuanensis* (si.chuan.en’sis. N.L. fem. adj. *sichuanensis*, referring to Sichuan Province, China, where the type strain was recovered). The species status is determined using ANI, *is*DDH (Table 1 and Supplementary Dataset S1), core gene-based phylogenomic analysis (Figure 2) and phenotypic assays (see below).

Cells are Gram-stain-negative, facultatively anaerobic, motile, non-spore-forming and short-rod shaped (0.5–0.8 μm wide and 1.0–2.0 μm long, Supplementary Figure S1). Colonies growing on the nutrient agar after 12 h are round, smooth, convex, white. Growth occurs between 8 and 42°C, in the 0 to 5% NaCl (w/v), and at pH 5.0 to 9.0. Nitrate reduction, citrate utilization, and activities of lysine decarboxylase and ornithine decarboxylase are positive. It is able to assimilate esculin, amygdalin, arbulin, D-cellobiose, D-fructose, D-glucose, D-galactose, D-lactose, D-maltose, D-melibiose, D-sorbitol, D-trehalose, D-xylose, 5-keto-gluconate, L-arabinose, L-rhamnose, mannitol, malonate, methyl-α-D-glucopyranoside, *N*-acetylglucosamine, potassium gluconate, ribose, and salicin. It is negative for acetoin production (Voges–Proskauer), catalase, DNase, H_2_S production, indole production, oxidase and phenylalanine deaminase, and activities of arginine dihydrolase, β-galactosidase (ONPG), gelatinase, tryptophan deaminase and urease. It does not utilize adonitol, D-mannose, D-saccharose, dulcitol, erythritol, glycerol, inositol, raffinose and sucrose. The major cellular fatty acids are C_16:0_ (31.28%), C_17:0_ cyclo (17.56%) and summed in feature 8 (C_18:1_ω7c) (15.74%).

The type strain is 090646^T^ (also called SCKS090646^T^), recovered from a hospital sink in Chengdu, Sichuan province, China. Strain 090646^T^ has been deposited into Guangdong Microbiology Culture Center as GDMCC 1.1872^T^ and into Korean Collection for Type Cultures as KCTC 82166^T^. The draft genome of the type strain is 5,476,810 bp with a 54.51 mol% G + C content (DDBJ/EMBL/GenBank accession no. JABBJF000000000).

### Description of *Kluyvera* Genomospecies 1

As delineation of the strains could be determined using ANI and *is*DDH (Table 3 and Supplementary Dataset S1) as well as core gene-based phylogenomic analysis (Figure 3), it is proposed to designate a novel genomospecies, *Kluyvera* genomospecies 1, represented by strain L2 (GenBank accession no. LGHZ00000000 for the genome sequence).

### Description of *Kluyvera* Genomospecies 2

As delineation of the strains could be determined using ANI and *is*DDH (Table 3 and Supplementary Dataset S1) as well as core gene-based phylogenomic analysis (Figure 3), it is proposed to designate a novel genomospecies, *Kluyvera* genomospecies 2, represented by strain KA2 (GenBank accession no. PYHO00000000 for the genome sequence).

### Description of *Kluyvera* Genomospecies 3

As delineation of the strains could be determined using ANI and *is*DDH (Table 3 and Supplementary Dataset S1) as well as core gene-based phylogenomic analysis (Figure 3), it is proposed to designate a novel genomospecies, *Kluyvera* genomospecies 3, represented by strain PO2S7 (GenBank accession no. CP050321 for the genome sequence).

### Description of *Kluyvera* Genomospecies 4

As delineation of the strains could be determined using ANI and *is*DDH (Table 3 and Supplementary Dataset S1) as well as core gene-based phylogenomic analysis (Figure 3), it is proposed to designate a novel genomospecies, *Kluyvera* genomospecies 4, represented by strain D51-sc-1712206 (GenBank accession no. ERR2221162 for the genome sequence).

## Data Availability Statement

The datasets presented in this study can be found in online repositories. The names of the repository/repositories and accession number(s) can be found in the article/Supplementary Material.

## Author Contributions

ZZ designed the study. LL, YF, LW, and FQ performed the study. LL, YF, and ZZ analyzed the data. LL and ZZ drafted the manuscript. All authors contributed to approve the manuscript.

## Conflict of Interest

The authors declare that the research was conducted in the absence of any commercial or financial relationships that could be construed as a potential conflict of interest.

## Footnotes

1 http://www.eucast.org/

2 https://github.com/tseemann/abricate

3 http://genomicepidemiology.org/

4 https://github.com/tseemann/shovill

5 http://genomicepidemiology.org/

6 http://www.oligoevaluator.com
