# Corrigendum: Precise Species Identification and Taxonomy Update for the Genus *Kluyvera* With Reporting *Kluyvera sichuanensis* sp. nov.

**DOI:** 10.3389/fmicb.2020.615117

**Published:** 2020-12-08

**Authors:** Lina Liu, Yu Feng, Li Wei, Fu Qiao, Zhiyong Zong

**Affiliations:** ^1^Center of Infectious Diseases, West China Hospital, Sichuan University, Chengdu, China; ^2^Center for Pathogen Research, West China Hospital, Sichuan University, Chengdu, China; ^3^Division of Infectious Diseases, State Key Laboratory of Biotherapy, Chengdu, China; ^4^Department of Infection Control, West China Hospital, Sichuan University, Chengdu, China

**Keywords:** *Kluyvera sichuanensis*, *Kluyvera*, genome sequences, sinks, taxonomy

In the original article, there was an error in the Results and Description sections, which provided an incorrect accession number for the type strain deposited into the Guangdong Microbiology Culture Center (GDMCC).

A correction has been made to the **“Results”** section, which included an incorrect accession number for the deposited type strain. The subsection **“Strain identification”** should read as follows:

**Strain Identification**

Strain 090646^T^ was recovered from the residual water of a handwashing sink at an ICU in Chengdu, China, on April 2019 as part of an infection control surveillance program on sinks. The strain was preliminarily identified as *Kluyvera ascorbata* by MALDI-TOF. A nearly complete sequence (1,411 bp) of the 16S rRNA gene of strain 090646^T^ has the highest identity with that of *Kluyvera ascorbata* ATCC 33433^T^ (99.22%) and is also highly similar with those of *K. intermedia* NCTC 12125^T^ (98.88%), *K. cryocrescens* NBRC 102467^T^ (98.58%), *K. georgiana* ATCC 51603^T^ (98.30%), *Klebsiella aerogenes* KCTC 2190^T^ (98.23%), *Raoultella terrigena* ATCC 33257^T^ (98.09%), *Citrobacter braakii* ATCC 51113^T^ (97.95%), and *Lelliottia amnigena* NBRC 105700^T^ (97.94%). In a maximum-likelihood phylogenetic tree (Figure 1) based on the 16S rRNA gene sequence alignment, strain 090646^T^ was allocated within the *Kluyvera* clade. However, strain 090646^T^ forms a relatively long branch separating from other *Kluyvera* species (Figure 1) and it is well known that analysis on 16S rRNA gene sequence is insufficient for accurate bacterial species assignation (Mulet et al., 2020). We therefore performed whole genome sequencing by HiSeq X 10 (Illumina; San Diego, CA, United States) for strain 090646^T^. A total of 1.42 gigabyte bases were generated, which were then assembled into 117 contigs (*N50*, 144,979 bp). The draft genome of strain 090646^T^ is 5,476,810 bp with a 54.51 mol% G + C content, which has been deposited at DDBJ/EMBL/GenBank under the accession JABBJF000000000.

A total of 684 core genes (Supplementary Dataset S2 in the Supplementary file) were identified in genome sequences of type strains of all *Enterobacteriaceae* species (Supplementary Table S1). In the maximum-likelihood phylogenomic tree based on core genes (Figure 2), strain 090646^T^ was also allocated within the *Kluyvera* clade. The POCP values between strain 090646^T^ and type strains of all *Kluyvera* species ranged from 90.81 to 93.18% (Table 1), suggesting that strain 090646^T^ indeed belonged to the genus *Kluyvera*. The ANI values between strain 090646^T^ and type strains of all *Kluyvera* species ranged from 84.15 to 90.10% (Table 1), lower than the ≥95–95% cutoff for defining species (Richter and Rossello-Mora, 2009). Consistently, *is*DDH values between strain 090646^T^ and type strains of all *Kluyvera* species ranged from 28.2 to 42.3% (Table 1), which are well below the ≥70.0% cutoff to define species (Richter and Rossello-Mora, 2009; Meier-Kolthoff et al., 2013). Both ANI and *is*DDH analyses clearly suggest that strain 090646^T^ represents a novel species of the genus *Kluyvera*. We proposed the species name as *Kluyvera sichuanensis* (si.chuan.en'sis. N.L. adj. *sichuanensis*, referring to Sichuan Province, China, where the type strain was recovered) after phenotypic characterization (see below). The type strain 090646^T^ has been deposited into Guangdong Microbial Culture Collection Center as GDMCC 1.1872^T^ and into the Korean Collection for Type Cultures as KCTC82166^T^.

A correction has been made to the section **“Descriptions”**, which included an incorrect accession number for the deposited type strain. The subsection **“Description of *Kluyvera sichuanensis sp. nov.”*** should read as follows:

**Description of *Kluyvera sichuanensis* sp. nov**.

*Kluyvera sichuanensis* (si.chuan.en'sis. N.L. fem. adj. *sichuanensis*, referring to Sichuan Province, China, where the type strain was recovered). The species status is determined using ANI, *is*DDH (Table 1 and Supplementary Dataset S1), core gene-based phylogenomic analysis (Figure 2) and phenotypic assays (see below).

Cells are Gram-stain-negative, facultatively anaerobic, motile, non-spore-forming and short-rod shaped (0.5–0.8 μm wide and 1.0–2.0 μm long, Supplementary Figure S1). Colonies growing on the nutrient agar after 12 h are round, smooth, convex, white. Growth occurs between 8 and 42°C, in the 0 to 5% NaCl (w/v), and at pH 5.0 to 9.0. Nitrate reduction, citrate utilization, and activities of lysine decarboxylase and ornithine decarboxylase are positive. It is able to assimilate esculin, amygdalin, arbulin, D-cellobiose, D-fructose, D-glucose, D-galactose, D-lactose, D-maltose, D-melibiose, D-sorbitol, D-trehalose, D-xylose, 5-keto-gluconate, L-arabinose, L-rhamnose, mannitol, malonate, methyl-α-D-glucopyranoside, *N*-acetylglucosamine, potassium gluconate, ribose, and salicin. It is negative for acetoin production (Voges–Proskauer), catalase, DNase, H_2_S production, indole production, oxidase and phenylalanine deaminase, and activities of arginine dihydrolase, β-galactosidase (ONPG), gelatinase, tryptophan deaminase and urease. It does not utilize adonitol, D-mannose, D-saccharose, dulcitol, erythritol, glycerol, inositol, raffinose and sucrose. The major cellular fatty acids are C_16:0_ (31.28%), C_17:0_ cyclo (17.56%) and summed in feature 8 (C_18:1_ω7c) (15.74%).

The type strain is 090646^T^ (also called SCKS090646^T^), recovered from a hospital sink in Chengdu, Sichuan province, China. Strain 090646^T^ has been deposited into Guangdong Microbiology Culture Center as GDMCC 1.187**2**^T^ and into Korean Collection for Type Cultures as KCTC 82166^T^. The draft genome of the type strain is 5,476,810 bp with a 54.51 mol% G + C content (DDBJ/EMBL/GenBank accession no. JABBJF000000000).

The authors apologize for the error and state that this correction does not change the scientific conclusions of the article in any way. The original article has been updated.
