# Supplementary material for: Precise Species Identification and Taxonomy Update for the Genus Kluyvera With Reporting Kluyvera sichuanensis sp. nov
Source: Front Microbiol. 2020 Sep 16;11:579306. doi: 10.3389/fmicb.2020.579306 (PMC7524892; doi:10.3389/fmicb.2020.579306)

Fig S1. Electron microscope image of *Kluyvera sichuanensis* 090646^{T}. Cell morphology was examined by an H-7650 transmission electron microscope (Hitachi; Tokyo, Japan). Cells were 1.0–2.0 μm long and 0.5–0.8 μm wide in diameter.

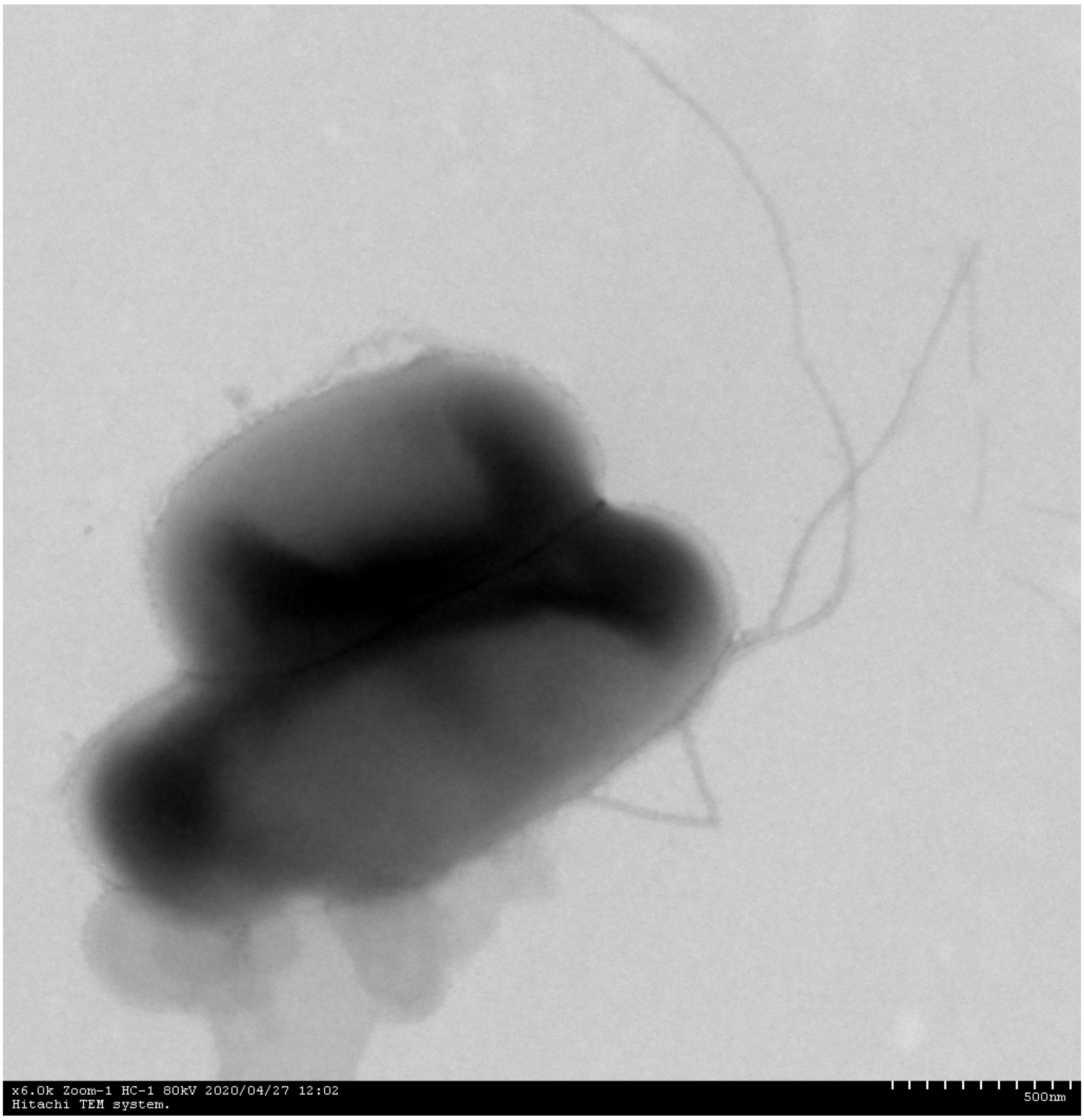

Supplement: Supplementary file 1 [file Image_1.PDF]
