# Supplementary material for: Precise Species Identification and Taxonomy Update for the Genus Kluyvera With Reporting Kluyvera sichuanensis sp. nov
Source: Front Microbiol. 2020 Sep 16;11:579306. doi: 10.3389/fmicb.2020.579306 (PMC7524892; doi:10.3389/fmicb.2020.579306)
Supplement: Supplementary file 2 [file Table_1.DOCX]

Table S1. Type strains of species within the family *Enterobacteriaceae*

| **Species** | **Type strain** | **Assembly accession** | **Assembly level** | **Genome size, bp** | **GC (%)** | **Coverage，x** | **No. of contigs** | **Completeness%** | **Contamination** |
| --- | --- | --- | --- | --- | --- | --- | --- | --- | --- |
| *[Erwinia] teleogrylli* | SCU-B244^T^ | GCA_001484765.1 | Scaffold | 5,453,996 | 55.33 | 106.00 | 132 | 99.14 | 0.07 |
| *[Kluyvera] intestini* | GT-16^T^ | GCA_001856865.3 | Contig | 5,784,959 | 53.01 | 150.00 | 57 | 100.00 | 0.14 |
| *Atlantibacter hermannii* | NBRC 105704^T^ | GCA_000248015.1 | Complete | 4,553,773 | 54.08 | 100.00 | 1 | 99.12 | 0.00 |
| *Atlantibacter subterranea* | DSM 16208^T^ | SRR3502980 | Contig | 4,752,424 | 55.08 | 97.47 | 105 | 100.00 | 0.00 |
| *Biostraticola tofi* | DSM 19580^T^ | GCA_004343195.1 | Contig | 4,290,872 | 53.86 | 349.00 | 32 | 99.45 | 0.83 |
| *Buttiauxella agrestis* | ATCC 33320^T^ | GCA_000735355.1 | Contig | 4,725,816 | 50.78 | 15.00 | 93 | 100.00 | 0.00 |
| *Buttiauxella brennerae* | ATCC 51605^T^ | GCA_001654925.1 | Contig | 4,758,272 | 50.63 | 38.00 | 88 | 100.00 | 0.00 |
| *Buttiauxella ferragutiae* | ATCC 51602^T^ | GCA_001654915.1 | Contig | 5,087,094 | 50.38 | 39.00 | 89 | 100.00 | 0.00 |
| *Buttiauxella gaviniae* | ATCC 51604^T^ | GCA_001654835.1 | Contig | 5,068,083 | 49.41 | 37.00 | 133 | 100.00 | 0.00 |
| *Buttiauxella izardii* | CCUG 35510^T^ | GCA_003601925.1 | Contig | 4,769,981 | 51.26 | 280.00 | 91 | 99.17 | 0.00 |
| *Buttiauxella noackiae* | ATCC 51607^T^ | GCA_001654865.1 | Contig | 4,803,576 | 49.49 | 35.00 | 104 | 100.00 | 0.28 |
| *Buttiauxella warmboldiae* | CCUG 35512^T^ | GCA_003818135.1 | Contig | 4,301,460 | 52.38 | 98.00 | 135 | 100.00 | 0.00 |
| *Cedecea colo* | ZA 0188^T^ | GCA_011808225.1 | Contig | 4,459,929 | 53.06 | 12.00 | 36 | 100.00 | 0.00 |
| *Cedecea davisae* | DSM 4568^T^ | GCA_000412335.2 | Scaffold | 4,886,981 | 54.35 | 38.90 | 44 | 100.00 | 0.00 |
| *Cedecea lapagei* | NCTC11466^T^ | GCA_900635955.1 | Complete | 4,778,444 | 54.81 | 100.00 | 1 | 100.00 | 0.00 |
| *Cedecea neteri* | NBRC 105707^T^ | GCA_001571265.1 | Contig | 5,203,266 | 54.11 | 109.00 | 139 | 100.00 | 0.83 |
| *Citrobacter amalonaticus* | NCTC10805^T^ | GCA_900460855.1 | Contig | 5,093,340 | 53.43 | 100.00 | 3 | 99.21 | 0.00 |
| *Citrobacter braakii* | ATCC 51113^T^ | GCA_002075345.1 | Contig | 5,575,578 | 51.9 | 100.00 | 107 | 100.00 | 0.00 |
| *Citrobacter europaeus* | 97/99^T^ | GCA_900079995.1 | Contig | 5,293,620 | 51.89 | 80.00 | 36 | 100.00 | 0.00 |
| *Citrobacter freundii* | ATCC 8090^T^ | GCA_011064845.1 | Complete | 4,957,773 | 51.66 | 269.00 | 1 | 100.00 | 0.00 |
| *Citrobacter gillenii* | CIP 106783^T^ | ERR664249 | Contig | 4,878,433 | 52.58 | 117.37 | 79 | 100.00 | 0.00 |
| *Citrobacter koseri* | NCTC10786^T^ | GCA_900446925.1 | Contig | 4,661,458 | 53.81 | 100.00 | 11 | 93.62 | 0.00 |
| *Citrobacter murliniae* | CIP 104556^T^ | ERR664250 | Contig | 4,938,656 | 50.79 | 120.64 | 30 | 100.00 | 0.08 |
| *Citrobacter pasteurii* | CIP 55.13^T^ | GCA_000826205.1 | Contig | 4,986,623 | 51.8 | 83.00 | 56 | 100.00 | 0.00 |
| *Citrobacter portucalensis* | A60^T^ | GCA_002042885.1 | Contig | 4,916,306 | 52.01 | 100.00 | 40 | 100.00 | 0.00 |
| *Citrobacter rodentium* | NBRC 105723^T^ | GCA_000759815.1 | Contig | 5,186,777 | 54.63 | 63.00 | 139 | 100.00 | 0.00 |
| *Citrobacter sedlakii* | NBRC 105722^T^ | GCA_000759835.1 | Contig | 4,631,466 | 54.72 | 103.00 | 30 | 100.00 | 0.00 |
| *Citrobacter werkmanii* | NBRC 105721^T^ | GCA_000759755.1 | Contig | 4,947,997 | 52.07 | 125.00 | 30 | 100.00 | 0.00 |
| *Citrobacter youngae* | NCTC13709^T^ | GCA_900638065.1 | Complete | 4,867,355 | 51.8 | 100.00 | 1 | 100.00 | 0.28 |
| *Cronobacter condimenti* | LMG 26250^T^ | GCA_001277255.1 | Complete | 4,499,482 | 55.78 | 321.00 | 2 | 100.00 | 0.21 |
| *Cronobacter dublinensis* | LMG 23823^T^ | GCA_001277235.1 | Complete | 4,628,405 | 57.8 | 97.00 | 2 | 99.94 | 0.21 |
| *Cronobacter malonaticus* | LMG 23826^T^ | GCA_001277215.2 | Complete | 4,473,761 | 56.85 | 250.00 | 3 | 99.94 | 0.28 |
| *Cronobacter muytjensii* | ATCC 51329^T^ | GCA_001277195.1 | Complete | 4,364,114 | 57.69 | 196.00 | 1 | 100.00 | 0.21 |
| *Cronobacter sakazakii* | ATCC 29544^T^ | GCA_000982825.1 | Complete | 4,663,565 | 56.64 | 73.00 | 4 | 99.94 | 0.00 |
| *Cronobacter turicensis* | z3032^T^ | GCA_000027065.2 | Complete | 4,599,092 | 57.25 | N/A | 4 | 99.94 | 0.21 |
| *Cronobacter universalis* | NCTC 9529^T^ | GCA_001277175.1 | Complete | 4,436,873 | 57.83 | 191.00 | 2 | 99.94 | 0.21 |
| *Edaphovirga cremea* | DSM 105170^T^ | GCA_003332275.1 | Scaffold | 5,193,810 | 50.33 | 100.00 | 70 | 100.00 | 0.00 |
| *Enterobacillus tribolii* | DSM 103736^T^ | GCA_003363015.1 | Scaffold | 4,931,017 | 56.03 | 190.00 | 39 | 100.00 | 0.83 |
| *Enterobacter asburiae* | ATCC 35953^T^ | GCA_001521715.1 | Complete | 4,806,219 | 55.47 | 85.08 | 5 | 100.00 | 0.00 |
| *Enterobacter bugandensis* | EB-247^T^ | GCA_900324475.1 | Complete | 4,717,613 | 56 | 30.00 | 1 | 100.00 | 0.00 |
| *Enterobacter cancerogenus* | ATCC 33241^T^ | GCA_900185905.1 | Contig | 4,879,939 | 55.63 | 45.00 | 142 | 100.00 | 0.83 |
| *Enterobacter chengduensis* | WCHECh050004^T^ | GCA_001984825.2 | Complete | 5,218,270 | 55.74 | 200.00 | 2 | 100.00 | 0.00 |
| *Enterobacter chuandaensis* | 90028^T^ | GCA_003594915.1 | Contig | 4,662,901 | 55.67 | 200.00 | 38 | 100.00 | 0.86 |
| *Enterobacter cloacae* | ATCC 13047^T^ | GCA_000025565.1 | Complete | 5,598,796 | 54.58 | N/A | 3 | 100.00 | 0.00 |
| *Enterobacter hoffmannii* | DSM 14563^T^ | GCA_001729745.1 | Complete | 4,678,566 | 55.33 | 15.31 | 2 | 100.00 | 0.41 |
| *Enterobacter hormaechei* | ATCC 49162^T^ | GCA_001875655.1 | Scaffold | 4,890,213 | 55.17 | 68.00 | 4 | 100.00 | 0.41 |
| *Enterobacter huaxiensis* | WCHEHu090008^T^ | GCA_003594935.1 | Contig | 5,037,445 | 55.71 | 200.00 | 45 | 100.00 | 0.00 |
| *Enterobacter kobei* | DSM 13645^T^ | GCA_001729765.1 | Complete | 4,927,478 | 54.91 | 11.97 | 2 | 100.00 | 0.83 |
| *Enterobacter lignolyticus* | SCF1^T^ | GCA_000164865.1 | Complete | 4,814,049 | 57.02 | 30.00 | 1 | 99.92 | 0.00 |
| *Enterobacter ludwigii* | EN-119^T^ | GCA_001750725.1 | Complete | 4,952,770 | 54.6 | 69.00 | 2 | 100.00 | 0.83 |
| *Enterobacter mori* | LMG 25706^T^ | GCA_000211415.1 | Scaffold | 4,960,219 | 55.3 | 200.00 | 139 | 98.14 | 1.65 |
| *Enterobacter oligotrophica* | CCA6^T^ | GCA_009176645.1 | Complete | 4,476,585 | 54.3 | 330.00 | 1 | 100.00 | 1.65 |
| *Enterobacter roggenkampii* | DSM 16690^T^ | GCA_001729805.1 | Complete | 4,899,997 | 56.04 | 13.10 | 2 | 100.00 | 0.83 |
| *Enterobacter sichuanensis* | WCHECL1597^T^ | GCA_002939185.1 | Contig | 4,938,969 | 55.21 | 200.00 | 204 | 100.00 | 0.00 |
| *Enterobacter soli* | ATCC BAA-2102^T^ | GCA_001654845.1 | Contig | 4,960,767 | 53.76 | 40.00 | 74 | 100.00 | 0.83 |
| *Enterobacter xiangfangensis* | LMG27195^T^ | GCA_001729785.1 | Complete | 4,661,849 | 55.28 | 16.15 | 1 | 99.59 | 0.41 |
| *Escherichia albertii* | Albert 19982^T^ | ERR162699 | Contig | 4,448,618 | 49.74 | 59.68 | 285 | 100.00 | 0.00 |
| *Escherichia coli* | ATCC 11775^T^ | GCA_003697165.2 | Complete | 5,034,834 | 50.64 | 800.00 | 2 | 100.00 | 0.00 |
| *Escherichia fergusonii* | ATCC 35469^T^T | GCA_000026225.1 | Complete | 4,643,861 | 49.92 | N/A | 2 | 100.00 | 0.83 |
| *Escherichia marmotae* | HT073016^T^ | GCA_002900365.1 | Complete | 4,896,291 | 50.4 | 83.00 | 3 | 100.00 | 0.00 |
| *Franconibacter helveticus* | LMG 23732^T^ | GCA_000463115.2 | Scaffold | 4,521,165 | 56.01 | 71.00 | 56 | 99.94 | 0.00 |
| *Franconibacter pulveris* | DSM 19144^T^ | GCA_000621185.1 | Scaffold | 4,710,926 | 56.56 | N/A | 36 | 100.00 | 0.21 |
| *Gibbsiella quercinecans* | FRB97^T^ | GCA_002291425.1 | Complete | 5,548,506 | 55.94 | 400.00 | 1 | 99.59 | 0.83 |
| *Izhakiella australiensis* | D4N98^T^ | GCA_002006995.1 | Scaffold | 5,015,434 | 54.04 | 110.00 | 61 | 100.00 | 0.00 |
| *Izhakiella capsodis* | N6PO6^T^ | GCA_900115045.1 | Scaffold | 3,508,271 | 50.1 | 322.00 | 30 | 100.00 | 0.00 |
| *Klebsiella aerogenes* | KCTC 2190^T^ | GCA_000215745.1 | Complete | 5,280,350 | 54.85 | N/A | 1 | 100.00 | 0.00 |
| *Klebsiella africana* | CIP 111653^T^ | GCA_900978845.1 | Contig | 5,156,720 | 57.31 | 206.00 | 50 | 99.17 | 0.00 |
| *Klebsiella grimontii* | 06D021^T^ | GCA_900200035.1 | Contig | 6,168,876 | 55.37 | 56.00 | 54 | 100.00 | 0.05 |
| *Klebsiella huaxiensis* | WCHKl090001^T^ | GCA_003261575.2 | Complete | 6,301,221 | 53.28 | 200.00 | 2 | 100.00 | 0.00 |
| *Klebsiella michiganensis* | DSM 25444^T^ | GCA_002925905.1 | Scaffold | 6,193,009 | 55.97 | 225.00 | 32 | 100.00 | 0.00 |
| *Klebsiella oxytoca* | NBRC 105695^T^ | GCA_001598695.1 | Contig | 5,793,098 | 55.14 | 105.00 | 43 | 100.00 | 0.00 |
| *Klebsiella pasteurii* | CIP 111696^T^ | GCA_902158725.1 | Scaffold | 6,006,767 | 55.33 | 100.00 | 23 | 100.00 | 0.00 |
| *Klebsiella pneumoniae* | FDAARGOS_775^T^ | GCA_006364295.1 | Complete | 5,573,867 | 56.98 | 925.17 | 5 | 100.00 | 0.96 |
| *Klebsiella quasipneumoniae* | 01A030^T^ | GCA_000751755.1 | Contig | 5,465,736 | 57.96 | 137.00 | 65 | 99.97 | 0.00 |
| *Klebsiella quasivariicola* | KPN1705^T^ | GCA_002269255.1 | Complete | 5,946,706 | 56.66 | 88.00 | 4 | 100.00 | 0.00 |
| *Klebsiella spallanzanii* | CIP 111695^T^ | GCA_902158555.1 | Scaffold | 6,186,380 | 53.3 | 100.00 | 47 | 100.00 | 0.55 |
| *Klebsiella variicola* | DSM 15968^T^ | GCA_000828055.2 | Complete | 5,521,203 | 57.56 | 185.00 | 1 | 100.00 | 0.00 |
| *Kluyvera ascorbata* | ATCC 33433^T^ | GCA_000735365.1 | Contig | 4,932,354 | 54.34 | 9.00 | 253 | 99.17 | 1.31 |
| *Kluyvera cryocrescens* | NBRC 102467^T^ | GCA_001571285.1 | Contig | 5,044,663 | 53.85 | 122.00 | 101 | 100.00 | 0.00 |
| *Kluyvera georgiana* | ATCC 51603^T^ | GCA_001654985.1 | Contig | 5,068,984 | 54.53 | 34.00 | 163 | 100.00 | 1.45 |
| *Kluyvera intermedia* | NCTC12125^T^ | GCA_900635475.1 | Complete | 4,739,056 | 52.58 | 100.00 | 1 | 98.00 | 0.00 |
| *Kosakonia arachidis* | Ah-143^T^ | GCA_900116535.1 | Scaffold | 5,135,597 | 52.54 | 195.00 | 23 | 100.00 | 0.41 |
| *Kosakonia cowanii* | 888-76^T^ | GCA_001975225.1 | Complete | 4,857,324 | 56.22 | 80.00 | 3 | 100.00 | 0.00 |
| *Kosakonia oryzae* | Ola 51^T^ | GCA_001658025.1 | Complete | 5,303,342 | 54.01 | 128.00 | 1 | 95.04 | 0.41 |
| *Kosakonia oryzendophytica* | REICA 082^T^ | GCA_900094925.1 | Scaffold | 4,841,527 | 53.73 | 262.00 | 32 | 100.00 | 0.41 |
| *Kosakonia oryziphila* | REICA 142^T^ | GCA_900094795.1 | Scaffold | 4,814,900 | 52.75 | 301.00 | 129 | 100.00 | 0.41 |
| *Kosakonia pseudosacchari* | JM-387^T^ | GCA_900184035.1 | Contig | 4,956,546 | 53.9 | 109.00 | 29 | 99.97 | 0.00 |
| *Kosakonia quasisacchari* | WCHKQ120001^T^ | GCA_004331415.1 | Contig | 5,149,797 | 53.33 | 200.00 | 106 | 99.97 | 0.41 |
| *Kosakonia radicincitans* | DSM 16656^T^ | GCA_000280495.2 | Complete | 6,122,453 | 53.7 | N/A | 3 | 100.00 | 1.03 |
| *Kosakonia sacchari* | SP1^T^ | GCA_000300455.4 | Complete | 4,902,027 | 53.74 | 63.00 | 1 | 99.97 | 0.00 |
| *Leclercia adecarboxylata* | ATCC 23216^T^ | GCA_000735515.1 | Contig | 4,991,160 | 55.57 | 18.00 | 169 | 100.00 | 0.00 |
| *Lelliottia amnigena* | NCTC12124^T^ | GCA_900635465.1 | Complete | 4,471,442 | 52.86 | 100.00 | 1 | 98.69 | 0.83 |
| *Lelliottia jeotgali* | PFL01^T^ | GCA_002271215.1 | Complete | 4,603,334 | 54.24 | 209.00 | 1 | 100.00 | 0.00 |
| *Lelliottia nimipressuralis* | CICC 24156^T^ | GCA_008244655.1 | Contig | 4,649,469 | 54.89 | 200.00 | 44 | 100.00 | 0.00 |
| *Limnobaculum parvum* | HYN0051^T^ | GCA_003096015.2 | Complete | 3,841,771 | 46.04 | 202.50 | 1 | 99.59 | 0.00 |
| *Mangrovibacter phragmitis* | MP23^T^ | GCA_001655675.1 | Contig | 4,947,475 | 49.91 | 309.00 | 50 | 100.00 | 0.41 |
| *Mangrovibacter plantisponsor* | DSM 19579^T^ | GCA_003182475.1 | Scaffold | 5,352,990 | 50.43 | 245.00 | 56 | 100.00 | 0.69 |
| *Metakosakonia massiliensis* | JC163^T^ | GCA_000321045.2 | Scaffold | 5,003,540 | 55.48 | N/A | 236 | 98.66 | 0.83 |
| *Pluralibacter gergoviae* | NBRC 105706^T^ | GCA_001598855.1 | Contig | 5,662,775 | 58.64 | 96.00 | 104 | 99.81 | 0.83 |
| *Pluralibacter pyrinus* | ATCC 49851^T^ | SRR6219938 | Contig | 4,953,163 | 57.37 | 141.12 | 41 | 99.97 | 0.96 |
| *Pseudescherichia vulneris* | NBRC 102420^T^ | GCA_000759795.1 | Contig | 4,374,581 | 56.41 | 130.00 | 55 | 100.00 | 0.00 |
| *Pseudocitrobacter faecalis* | DSM 27453^T^ | GCA_003315335.1 | Scaffold | 5,155,726 | 53.02 | 94.00 | 49 | 100.00 | 0.00 |
| *Pseudocitrobacter vendiensis* | CPO20170097^T^ | ERR3255970 | Contig | 5,418,146 | 53.13 | 155.05 | 146 | 100.00 | 0.00 |
| *Pseudenterobacter timonensis* | mt20^T^ | GCA_900021175.1 | Contig | 4,199,690 | 56.81 | 106.00 | 13 | 100.00 | 0.00 |
| *Raoultella electrica* | DSM 102253^T^ | GCA_006711645.1 | Complete | 5,785,203 | 55.06 | 130.00 | 6 | 100.00 | 0.62 |
| *Raoultella ornithinolytica* | NBRC 105727^T^ | GCA_001598295.1 | Contig | 5,533,930 | 55.65 | 118.00 | 35 | 100.00 | 0.00 |
| *Raoultella planticola* | ATCC 33531^T^ | GCA_000735435.1 | Contig | 5,668,028 | 55.75 | 14.00 | 83 | 99.79 | 0.41 |
| *Raoultella terrigena* | NBRC 14941^T^ | GCA_006539725.1 | Contig | 5,515,279 | 57.44 | 105.00 | 55 | 99.97 | 0.00 |
| *Rosenbergiella nectarea* | 8N4^T^ | GCA_900111105.1 | Scaffold | 3,294,717 | 47.46 | 262.00 | 30 | 100.00 | 0.83 |
| *Salmonella bongori* | NCTC 12419^T^ | GCA_000252995.1 | Complete | 4,460,105 | 51.33 | N/A | 1 | 100.00 | 0.00 |
| *Salmonella enterica* | LT2^T^ | GCA_000006945.2 | Complete | 4,951,383 | 52.24 | N/A | 2 | 100.00 | 0.00 |
| *Scandinavium goeteborgense* | CCUG 66741^T^ | GCA_003935895.2 | Complete | 4,713,964 | 54.31 | 1000.00 | 2 | 97.28 | 0.83 |
| *Shimwellia blattae* | DSM 4481^T^ | GCA_000262305.1 | Complete | 4,158,725 | 56.51 | N/A | 1 | 99.94 | 0.00 |
| *Siccibacter colletis* | 1383^T^ | GCA_000696575.1 | Scaffold | 4,255,520 | 57.09 | 16.00 | 45 | 100.00 | 0.00 |
| *Siccibacter turicensis* | LMG 23730^T^ | GCA_000463155.2 | Scaffold | 4,236,729 | 57.85 | 48.00 | 74 | 100.00 | 0.28 |
| *Superficieibacter electus* | BP-1^T^ | GCA_002915575.1 | Contig | 5,680,147 | 52.4 | 70.00 | 89 | 100.00 | 0.00 |
| *Trabulsiella guamensis* | ATCC 49490^T^ | GCA_000734965.1 | Contig | 4,929,751 | 53.59 | 14.00 | 278 | 100.00 | 1.03 |
| *Yokenella regensburgei* | NCTC11966^T^ | GCA_900460805.1 | Contig | 4,937,525 | 54.75 | 100.00 | 2 | 100.00 | 0.83 |
