# Supplementary material for: Precise Species Identification and Taxonomy Update for the Genus Kluyvera With Reporting Kluyvera sichuanensis sp. nov
Source: Front Microbiol. 2020 Sep 16;11:579306. doi: 10.3389/fmicb.2020.579306 (PMC7524892; doi:10.3389/fmicb.2020.579306)
Supplement: Supplementary file 3 [file Table_2.DOCX]

Table S2. MICs of antimicrobial agents for strain 090646^T^

| **Antimicrobial agent** | **MIC, mg/L** | **Category** |
| --- | --- | --- |
| Amikacin | 4 | S |
| Ampicillin | >512 | R |
| Ampicillin-sulbactam | >512/256 | R |
| Aztreonam | 256 | R |
| Cefepime | 128 | R |
| Cefotaxime | 512 | R |
| Ceftazidime | >512 | R |
| Ceftriaxone | >512 | R |
| Cefuroxime | >512 | R |
| Ciprofloxacin | 8 | R |
| Chloramphenicol | 4 | S |
| Colistin | 2 | S |
| Imipenem | 16 | R |
| Meropenem | 64 | R |
| Piperacillin-tazobactam | 512/4 | R |
| Sulfamethoxazole-trimethoprim | 4.75/0.25 | S |
| Tigecycline | 2 | S |
