# Supplementary material for: Precise Species Identification and Taxonomy Update for the Genus Kluyvera With Reporting Kluyvera sichuanensis sp. nov
Source: Front Microbiol. 2020 Sep 16;11:579306. doi: 10.3389/fmicb.2020.579306 (PMC7524892; doi:10.3389/fmicb.2020.579306)
Supplement: Supplementary file 4 [file Table_3.DOCX]

Table S3. The length of *fes* in type strains of *Kluyvera* species

| Species | Length, bp |
| --- | --- |
| *Kluyvera georgiana* | 1,206 |
| *Kluyvera* genomosp. 3 | 1,206 |
| *Kluyvera ascorbata* | 1,209 |
| *Kluyvera sichuanensis* | 1,215 |
| *Kluyvera* genomosp. 2 | 1,215 |
| *Kluyvera cryocrescens* | 1,257 |
| *Kluyvera intermedia* | 1,257 |
| *Kluyvera* genomosp. 1 | 1,257 |
| *Kluyvera* genomosp. 4 | 1,257 |
